# *POLE* mutations improve the prognosis of endometrial cancer via regulating cellular metabolism through AMF/AMFR signal transduction

**DOI:** 10.1186/s12881-019-0936-2

**Published:** 2019-12-21

**Authors:** Yiran Li, Yiding Bian, Kai Wang, Xiao-Ping Wan

**Affiliations:** 1 Department of Gynecology, Shanghai First Maternity and Infant Hospital, Tongji University School of Medicine, 2699 Gaoke West Road, Shanghai, China; 2 Clinical and Translational Research Center, Shanghai First Maternity and Infant Hospital, Tongji University School of Medicine, Shanghai, China

**Keywords:** Endometrial cancer, Polymerase ɛ (POLE) mutation, Glucose metabolism, Autocrine motility factor (AMF), Prognosis

## Abstract

**Background:**

The morbidity and mortality of endometrial tumors, a common type of malignant cancer in women, have increased in recent years. POLE encodes the DNA polymerase ε, which is responsible for the leading strand DNA replication. Somatic mutations of POLE have been acknowledged in numerous cancers, resulting in the accumulation of DNA errors, leading to ultra-mutated tumors. Mutations in the exonuclease domain of POLE have been reported to improve progression-free survival in endometrial cancer. However, the potential relationship and underlying mechanism between POLE mutations and the prognosis of endometrial cancer patients remains unclear.

**Methods:**

The whole exome sequencing data, RNA sequencing data, and clinical information were obtained from the TCGA database and employed for the analyses in this study. The detailed mutational information was analyzed using whole exome sequencing data and the mutated genes were shown with OncoPlot. The survival curves and cox proportional hazards regression analysis were used to accessed patient prognosis, the association of clinical characteristics and prognosis. Differentially expressed genes were analyzed by the edgeR R/Bioconductor package, then the GSEA Pre-ranked tool was used for Gene Set Enrichment Analysis (GSEA) to estimate the function of genes. Expression values were clustered using hierarchical clustering with Euclidean distance and ward linkage by the dendextend R package.

**Results:**

POLE mutational status was proven to be an independent prognostic factor for endometrial cancer patients. Patients with somatic POLE mutations presented a favorable prognosis. POLE mutations regulated glycolysis and cytokine secretion, affecting cell metabolism and immune response. Autocrine motility factor (AMF)/PGI and AMFR/gp78 exhibited higher expression levels in POLE mutant patients. The comprehensive high expressions of AMFR/gp78 and low expression of POLE were associated with the favorable prognosis of endometrial cancer patients.

**Conclusions:**

This study showed the POLE mutations a vital factor in endometrial cancer patients, leading to a higher expression of AMF/PGI and AMFR/gp78. These results suggested comprehensive consideration of the POLE mutations, expression of AMF/PGI and AMFR/gp78 may provide a more feasible and effective approach for the treatment of endometrial cancer, which might improve the prognosis.

## Background

Endometrial cancer (EC), arising from the lining of the uterus, is one of the most common malignancy among women in developed countries. The incidence and mortality of endometrial cancer have risen in the United States in recent years, with estimated 74,200 new cases and 151,30 deaths in 2017, and about 76,470 new cases and 15,520 deaths in 2018 [1–4]. Although most patients present low-grade, early-stage diseases with favorable prognosis, the high-grade group accounts for a large proportion of deaths in endometrial cancer patients [5]. In addition, the mutation frequency profile was also related to different ethnicities and tumor grades in endometrial cancer patients [6]. PIK3CA, ARID1A, TP53, PIK3R1, and MUC16 are the most frequent mutations in endometrial cancer patients, and all of them have been reported to be associated with prognosis. PIK3CA, ARID1A, and MUC16 mutations were correlated with favorable prognosis, whereas TP53 and PIK3R1 mutations were correlated with poor prognosis [6, 7].

All cancers are caused by alterations in the DNA sequence including somatic mutations of the genomes in normal cells. DNA polymerases (Pols) are important enzymes involved in DNA replication and repair in vivo. According to the similarity of the protein sequence, Pols are categorized into six families: A, B, C, D, X and Y. Pols α, δ, and ε are three DNA polymerases essential during DNA replication in eukaryotes [8]. POLE (p261) encodes the DNA polymerase ε, which is a heterotetramer (p261, p59, p17, p12) [9] and responsible for the leading strand DNA replication [10, 11]. POLE contains the proof-reading exonuclease domain to ensure low mutation rates in replicating cells [12]. Early studies demonstrated that POLE mutations were observed in colorectal cancer, pancreatic cancer, ovarian cancer, ultra-mutated giant cell high-grade glioma, and endometrial cancer [13–17]. Germline mutations in the exonuclease domains of POLE were associated with a familial predisposition and increased risk of colorectal cancer and giant cell glioblastoma [18–20]. Somatic mutations in POLE have also been reported in these cancers [21]. These mutations lead to an accumulation of DNA errors during the replication process, resulting in ultra-mutated tumors associated with microsatellite stability [22].

Different forms of genomic instability in cancers are known to be associated with prognosis [23]. The somatic POLE mutations can lead to a genomically, histologically and clinically distinct subgroup of high-grade gliomas that are associated with a longer progression-free survival [16]. In endometrial cancer, somatic POLE proof-reading mutations were found in about 7% ECs, and the results have shown a tight association with high histologic grade [24]. Mutations in the exonuclease domain of POLE have been confirmed to be significantly associated with favorable prognosis and improved progression-free survival in endometrial cancer [25–27]. These results all suggest that POLE exonuclease domain mutations can serve as an important prognostic molecular marker associated with excellent outcomes and guide the management of endometrial cancer patients. The role of POLE and POLE mutants in carcinogenesis and the progression of endometrial carcinoma is not fully clear. In this study, we integrated whole exome sequencing data, patient clinical information, and the RNA sequencing data of the Uterine Corpus Endometrial Carcinoma (UCEC) project in the TCGA database for combinational analysis to elucidate the possible mechanism of POLE mutations on endometrial cancer.

## Methods

### TCGA data downloading

In this study, 530 endometrial cancer patients with whole exome sequencing data, 528 patients with clinical and survival information, and 526 patients with RNA expression data in the TCGA-UCEC project were downloaded from the TCGA database. In brief, the Mutation Annotation Format (MAF) data generated by the mutect2 algorithm from whole exome sequencing data, RNA sequencing raw counts (HTSeq-Counts) data, RNA sequencing FPKM data (HTSeq-FPKM), clinical and survival information were downloaded from the TCGA database through the TCGAbiolinks R/Bioconductor package [28].

### Analyses and visualization of somatic mutations

The detailed mutational information was extracted from the MAF file by the Maftools R/Bioconductor package [29]. Next, the plotmafSummary function was used to plot the summary of the MAF file for displaying the number of variant type and variant classification. The oncoplot function was used to plot the OncoPlot of the top 10 mutated genes and POLE. The lollipopPlot of POLE was plotted through the lollipopPlot function [29].

### Prognostic analyses

We used the Kaplan–Meier method and compared it with the log-rank test to depict the survival curves. Cox proportional hazards regression analysis was used for univariate and multivariate analyses to explore the association of clinical characteristics, POLE mutational status, and patient prognosis.

### Differentially expressed genes and enrichment analysis

The differentially expressed genes (DEGs) between the mutant and the wild type POLE endometrial cancer samples were analyzed by the edgeR R/Bioconductor package [30]. Gene ontology (GO) and the Kyoto Encyclopedia of Genes and Genomes (KEGG) were used for the enrichment analysis of the function and signaling pathway of the DEGs through the hypergeometric algorithm. The value of *p* < 0.05 was considered significantly enriched.

### Gene set enrichment analysis

Gene Set Enrichment Analysis (GSEA) was performed through the GSEA Pre-ranked tool in the GSEA software (http://www.broadinstitute.org/gsea/). The value of log_2_(FC) calculated by the edgeR package was used as the ranking metric. DEGs and all the other genes were both analyzed by GSEA. We used the C5 collection, which contains gene sets annotated by GO terms, and the C2 canonical pathway sub-collection contains 1329 gene sets that are annotated pathways mainly from the KEGG, BIOCARTA and REACTOME databases in the analysis.

### Hierarchical clustering

RNA sequencing FPKM data of 526 endometrial cancer samples in the TCGA database were used to assess the gene expression levels. Unsupervised hierarchical clustering was used to distinguish groups based on the expression pattern of target genes. Expression values of these genes in the 526 samples (where rows indicate the identity of the genes, columns indicate the identity of the patients) were clustered using hierarchical clustering with Euclidean distance and ward linkage by the dendextend R package [31] and heatmap.2 function in the gplots R package.

## Results

### POLE frequently mutates in endometrial Cancer

In this study, 530 endometrial cancer patients containing the whole exome sequencing data in the TCGA-UCEC project were included for subsequent analysis.

**Fig. 1:**
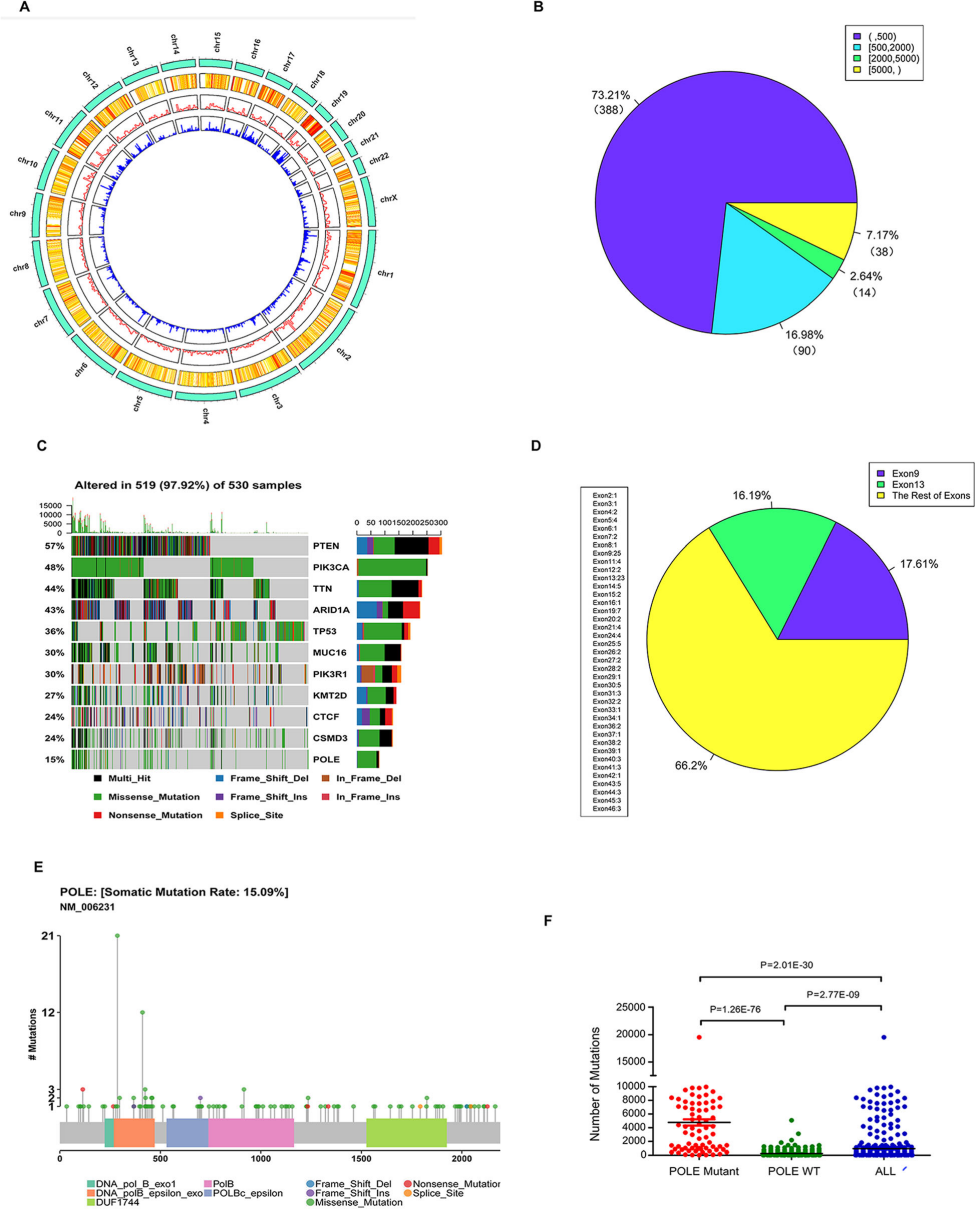
*POLE* frequently mutates in endometrial cancer. Somatic mutations were analyzed from the exome sequencing data of 530 endometrial cancer samples in the TCGA-UCEC project. **a** The distribution of the somatic mutation number in the endometrial cancer samples. **b** Kaplan–Meier curve and log-rank test for endometrial cancer patients based on mutation number. **c** The number of mutations for all patients (*N* = 530), patients with *POLE* mutant (*N* = 80), and patients with wild type *POLE* (*N* = 450). The data were compared using the wilcox.test. The bars represent the mean and SEM. **d** The OncoPlot of the top 10 mutated genes and *POLE*. The upper barplot indicates the number of mutations per patient, while the right barplot shows the number of mutations per gene. The mutation types were added as annotations at the bottom. Variants annotated as Multi_Hit are genes that mutated more than once in the same sample. **e** The pie chart of mutations of *POLE* occurring in multiple exons. **f** The lollipopPlot of *POLE*. The amino acid axis is labeled for domain. The mutation types were added as annotations at the bottom

Somatic alterations on a multiscale were identified in the exome of each endometrial cancer case including single-nucleotide polymorphisms (SNPs), short insertions (INS), and short deletions (DEL). In all 530 exome sequencing data of endometrial cancer samples, 886,371 somatic mutations were identified based on consensus calls from the mutect2 mutation-detection algorithm. 73.21% of endometrial cancer patients (Number = 388) contained less than 500 somatic mutations, 16.98% (Number = 90) contained 500–2000 somatic mutations, 2.64% (Number = 14) contained 2000–5000 somatic mutations and 7.17% (Number = 38) contained more than 2000 somatic mutations (Fig. 1a). The survival curve of endometrial cancer patients based on the number of mutations (mutation number > 500 or < 500) showed that the endometrial cancer patients had an improved prognosis when they held more mutations (*P* = 0.00033) (Fig. 1b). The patients with POLE somatic mutations had a higher tumor mutational burden compared with patients without POLE mutations (*P* = 1.26e-76) (Fig. 1c). These results suggest that POLE mutations may be an important prognostic factor in patients with endometrial cancer. In these somatic mutations, the major type of variants were SNPs (Number = 821,967), which was mainly the missense mutation (Additional file 1: Table S1). Silent, intron, UTR, 5′ flank, 3′ flank, splice region, and other synonymous SNPs were filtered and only non-synonymous variants were selected for further analysis. In the non-synonymous variants, most variants were SNPs and the major mutation was missense mutation (Additional file 1: Figures S1A, B). The top 10 mutated genes in endometrial cancer were: *PTEN* (57%), *PIK3CA* (48%), *TTN* (44%), *ARID1A* (43%), *TP53* (36%), *MUC16* (30%), *PIK3R1* (30%), *KMT2D* (27%), *CTCF* (24%), and *CSMD3* (24%). In addition, 15% of endometrial cancer patients had POLE somatic mutations as well, where more than 90% of POLE mutations were missense mutations (Fig. 1d).

*POLE* was the 123rd most frequently mutated gene in endometrial cancer with a mutational frequency of 80 in the 530 tumor samples (15%) (Fig. 1b, Additional file 1: Table S2). We identified 144 mutations in the *POLE* gene including 140 SNPs, 3 INS, and 1 DEL. In detail, the 144 mutations included 131 missense mutations, 7 nonsense mutations, 3 Frame_Shift_Ins, 1 Frame_Shift_Del, and 2 Splice_Site mutations (Additional file 1: Figures S1C, D). Most SNPs in *POLE* were substitutions between guanine and adenine (G/A) (Additional file 1: Figure S1E). Except for the 2 Splice_Site mutations, 142 somatic mutations occurred in exon regions containing 39 exons and more than 33% mutations occurred in exon9 (17.6%) and exon13 (16.2%) (Fig. 1e). The remaining 66.2% mutations were relatively evenly distributed in the other 37 exons (all exons contained less than 5% mutations). Most of the *POLE* mutations were missense mutations and these mutations were equally distributed from the N-terminal to the C-terminal of the *POLE* protein (Fig. 1f).

### POLE mutations are associated with improved prognosis of endometrial Cancer patients

A total of 528 endometrial cancer patients with clinical and survival information, including histological type, histologic grade, clinical stage, age at initial pathologic diagnosis, hypertension, history of neoadjuvant treatment, and diabetes in the TCGA-UCEC project, were used in this series. The detailed clinical characteristics are listed in Table 1. To explore the relationship between the *POLE* mutations and the clinical characteristics, we employed the chi-square test to establish the relationship between the *POLE* mutations and histological type, histologic grade, clinical stage, and age at initial pathologic diagnosis. The results showed that *POLE* mutational status was significantly associated with the histological type (*P* = 0.0006766) and age at the initial pathologic diagnosis (*P* = 4.39e-05). No significant relationship was found between the *POLE* mutational status and the histologic grade or clinical stage (*P* > 0.05) (Additional file 1: Table S3).

**Table 1:** Clinical characteristics of patients with endometrial cancer

Clinical characteristics	Cases (%)
Histological type	
Endometrioid endometrial adenocarcinoma	395(74.81%)
Mixed serous and endometrioid	22(4.17%)
Serous endometrial adenocarcinoma	111(21.02%)
Histologic grade	
G1	97(18.37%)
G2+G3	431(81.63%)
Clinical stage	
I + II	381(72.16%)
III + IV	147(27.84%)
Age at initial pathologic diagnosis	
< 60	174(32.95%)
≥60	352(66.67%)
NA^a^	2(0.38%)
Hypertension	
YES	260(49.24%)
NO	178(33.71%)
NA	90(17.05%)
History of neo adjuvant treatment	
YES	2(0.38%)
NO	526(99.26%)
Diabetes	
YES	108(20.45%)
NO	300(56.82%)
NA	120(22.73%)

^a^ *NA* Not available

**Fig. 2:**
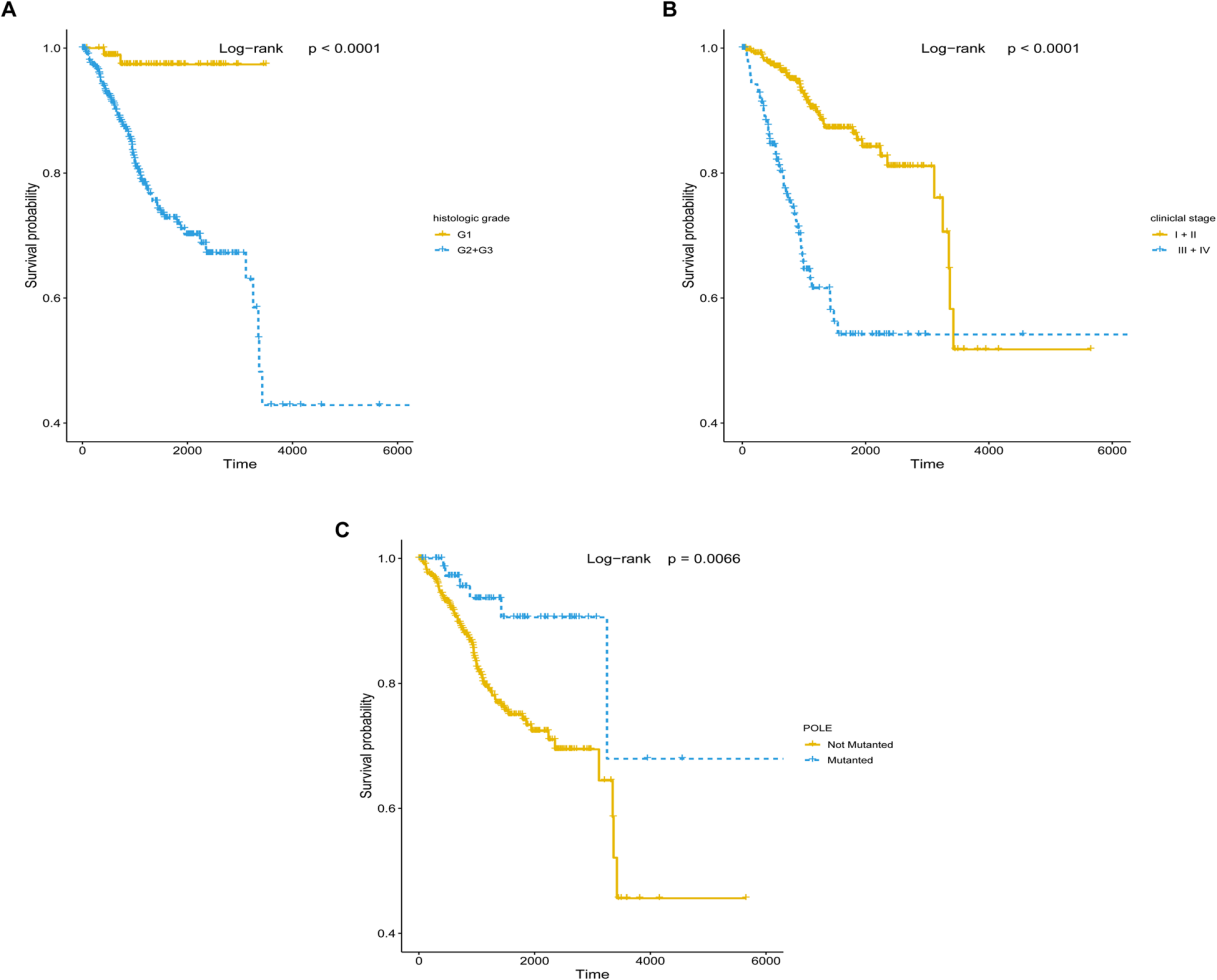
Histologic grade, clinical stage, and *POLE* mutational status are independent prognostic factors for endometrial cancer patients. **a** Kaplan–Meier curve and log-rank test for endometrial cancer patients based on histologic grade classification. **b** Kaplan–Meier curve and log-rank test for endometrial cancer patients based on clinical stage classification. **c** Kaplan–Meier curve and log-rank test for endometrial cancer patients based on *POLE* mutational status classification

The correlation between patient survival and histologic grade, clinical stage and POLE mutations, respectively, was analyzed by the Kaplan–Meier method and log-rank test. The results showed all three features correlate with patient survival with a *P* value less than 0.05 (Figs. 2a–c). Next, we conducted a Cox proportional hazards regression analysis to further investigate the correlation between patient overall survival and the four clinical features, POLE mutations, respectively. The *P* values of all five features were less than 0.05 in the univariate analysis, whereas, in the multivariate analysis, clinical stage (*P* = 2.50E-08), histologic grade (*P* = 0.00378), and POLE (*P* = 0.00194) mutational status were shown to be independent prognostic factors in endometrial cancer patients (Table 2). The results showed that endometrial cancer patients with low histologic grade (G1), early clinical stage (I+ II), and *POLE* mutations had favorable prognosis (Figs. 2a–c). Compared with the clinical stage, which showed the smallest *P* value in the Cox proportional hazards regression analysis and Kaplan–Meier curve in all three independent prognostic factors, histologic grade and *POLE* mutations were good stratified factors because it did not intersect in their survival curves.

**Table 2:** Univariate and multivariate Cox regression analysis in endometrial cancer patients

Clinical characteristics	Univariate Cox regression analysis			Multivariate Cox regression analysis		
	exp (coef)	*P*	95%CI	exp (coef)	*P*	95%CI
Histological type	1.661	6.26e-06	1.333 to 2.070	1.064	0.62231	0.831 to 1.363
Histologic grade (G1 vs. G2 + G3)	1.116	0.00076	1.047 to 1.189	1.1	0.00378	1.031 to 1.173
Clinical stage (I + II vs. III + IV)	1.063	5.38e-10	1.043 to 1.084	1.062	2.50e-08	1.040 to 1.085
Age at initial pathologic diagnosis (< 60 vs. ≥ 60)	1.803	0.0158	1.117 to 2.910	1.489	0.12497	0.895 to 2.478
*POLE* (WT vs. mutated)	0.334	0.00968	0.146 to 0.767	0.256	0.00194	0.108 to 0.606

Previous results suggest that *POLE* mutational status was associated with tumor mutational burden in endometrial cancers [32] consistent with our finding (Fig. 1c). To a certain extent, it is also possible that the hypermutated status might affect the prognosis of endometrial cancer patients. In order to increase the accuracy of the impact from *POLE* mutations and minimize the interference of hypermutated status on patient prognosis, we excluded hypermutated phenotypes (more than 500 mutations per sample) and found that the total tumor mutational burden decreased significantly. The survival curve of endometrial cancer patients showed that the exclusion of patients with hypermutated phenotypes altered the effects of *POLE* mutations on patient prognosis, hence the *POLE* mutations were no longer significantly associated with improved prognosis (Additional file 1: Figure S2). The variation in the relationship between *POLE* mutations and prognosis may be due to the limited sample size of the *POLE* mutant after removing the hypermutated phenotypes (Number = 10). The effect of mutational burden could not be considered in this study due to the limited number of samples.

### POLE mutations are involved in cellular glucose metabolism

**Fig. 3:**
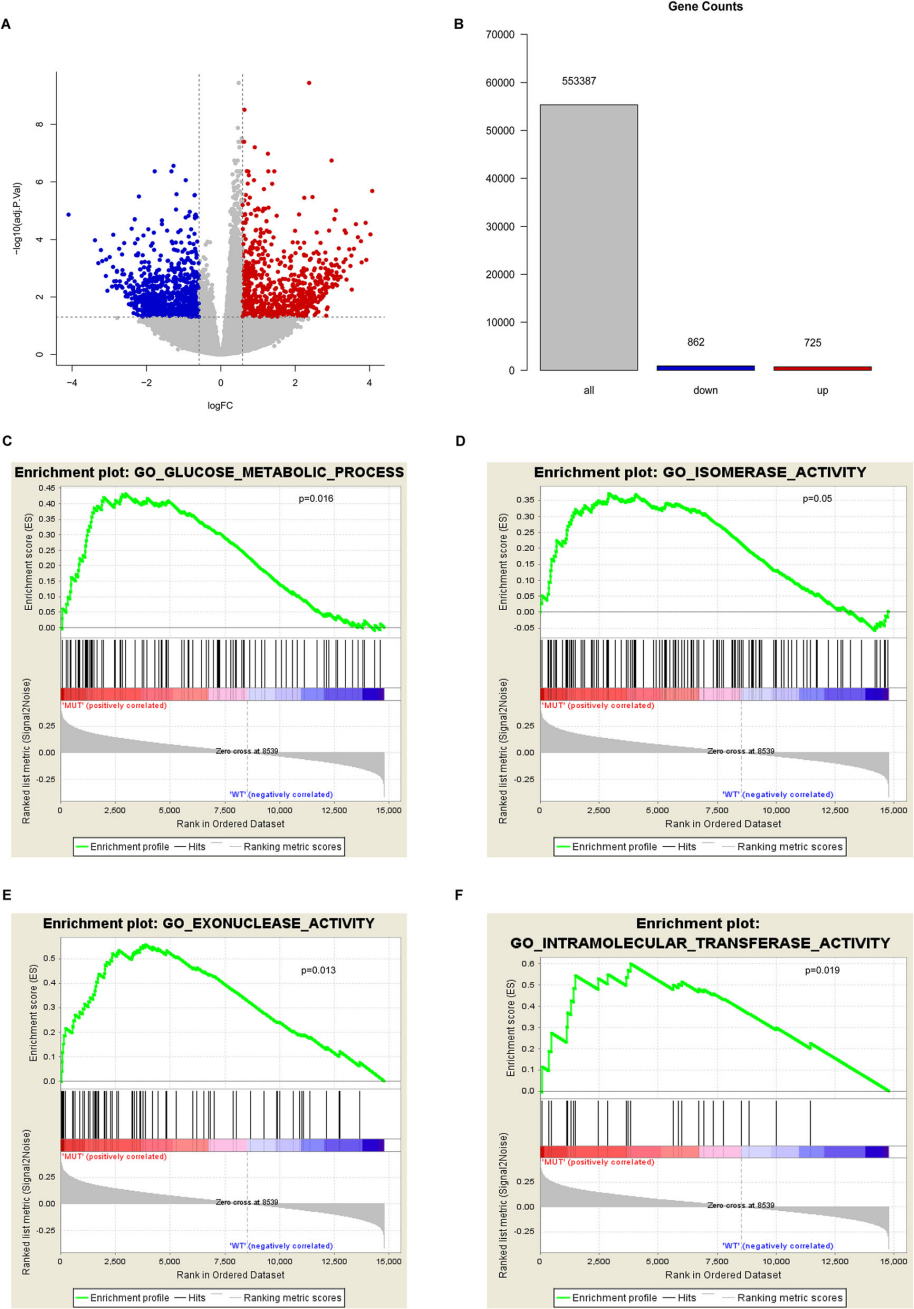
Mutated *POLE* is involved in glucose metabolism of endometrial cancer patients. **a** Volcano plot of differentially expressed genes (DEGs) between the mutant and wild type POLE endometrial cancer samples. X represents the foldchange, Y represents the *P* value. Red dot represents upregulated genes while the blue dot represents downregulated genes. **b**–**e** Gene Set Enrichment Analysis (GSEA) of the global gene expression profiling between mutant and wild type *POLE* endometrial cancer samples. Gene sets annotated by GO terms were used in the analysis. Gene set representing glucose metabolism (**c**), isomerase activity (**d**), exonuclease activity (**e**) and intramolecular transferase activity (**f**) are rich in POLE mutants

Endometrial cancer patients with *POLE* mutations had favorable prognoses, indicating that mutant *POLE* may play an important role in the development and progression of endometrial cancer. To further investigate the role of *POLE* mutations in endometrial cancer, we used transcriptome sequencing data from 526 patients to analyze the global gene expression profile and the differential expression between the two groups in 80 *POLE* mutant patients and 446 patients with wild type *POLE*. A total of 1587 differentially expressed genes (DEGs) were obtained according to the cut-off criteria (*P* < 0.05 and FC > 1.5) including 725 upregulated genes and 862 downregulated genes (Figs. 3a). To clarify the functional differences between these two groups, we conducted the gene set enrichment analysis (GSEA), which is a bioinformatics method that determines whether a set of terms shows statistically significant enrichment [33]. GSEA using Gene Ontology (GO) terms as the gene sets showed the enrichment in categories like glucose metabolic process, isomerase activity, exonuclease activity, and intramolecular transferase activity in endometrial cancer samples with *POLE* mutations (Figs. 3b–e). The T cell receptor signaling pathway and the production of molecular mediators of immune response were also significantly enriched in the GSEA results (Additional file 1: Figures S3A, B). GO analysis using the hypergeometric algorithm of 1587 DEGs was similar as those of the GSEA, which indicated that cytokine receptor activity, cytokine binding, cytokine receptor binding, chemokine receptor activity, and carbohydrate binding were enriched (Additional file 1: Figure S3C).

**Fig. 4:**
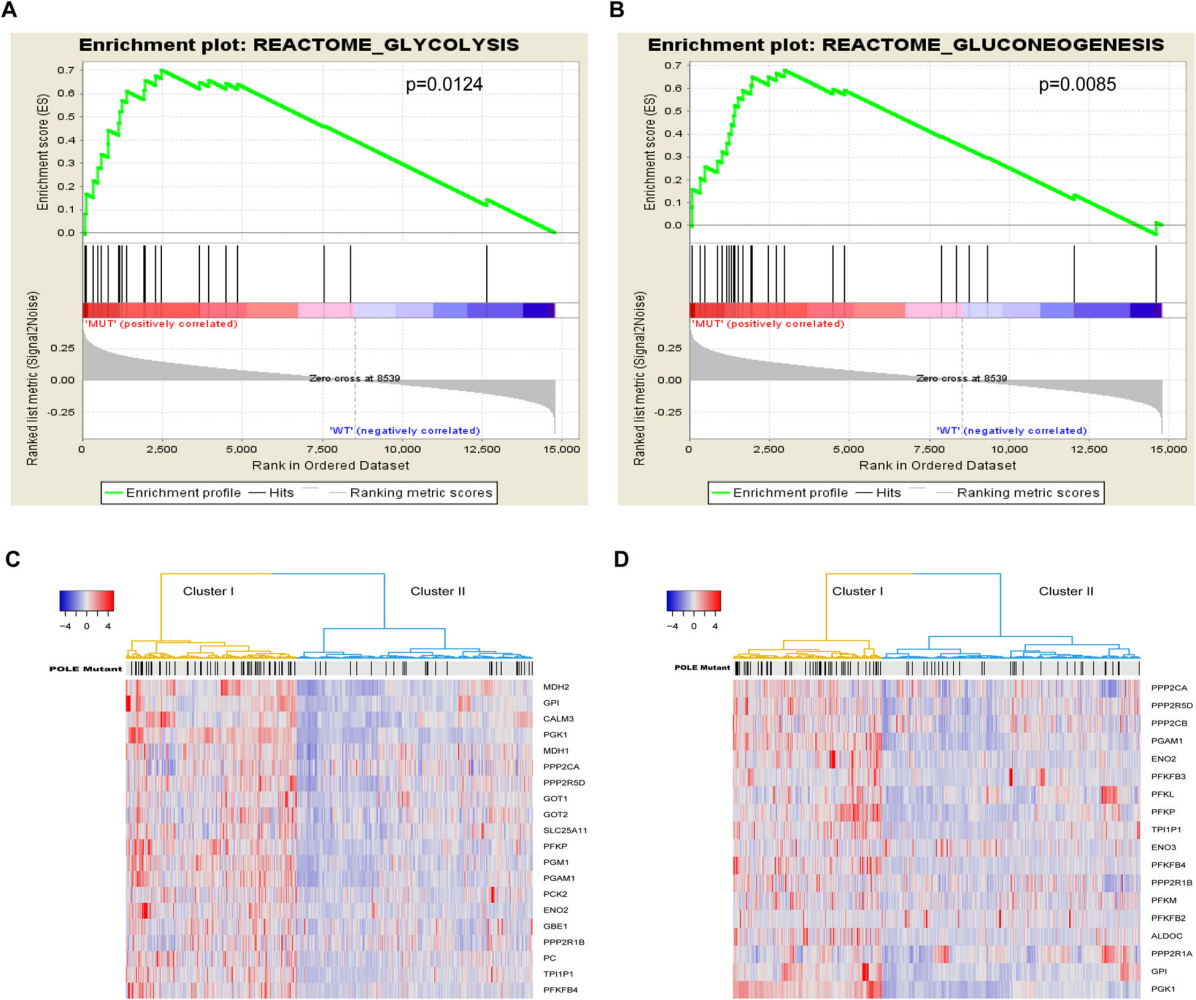
Cellular glucose metabolism was enriched in *POLE* mutants. **a**, **b** GSEA using the canonical pathways, which are annotated pathways mainly from the KEGG, BIOCARTA and REACTOME databases, as the gene sets in the analysis. Gene sets representing glycolysis (**a**) and gluconeogenesis (**b**) were enriched in the *POLE* mutants. **c** Unsupervised hierarchical clustering with Euclidean distance and ward linkage of expression matrixes from 20 genes inglycometabolis (**d**) Unsupervised hierarchical clustering with Euclidean distance and ward linkage of expression matrixes from 18 genes in glycolysis. The *POLE* mutational status for each tumor is depicted directly on top of each column. The black vertical lines represent the samples with *POLE* mutations. Red represents high gene expression, and blue represents relatively low gene expression

To further enlighten us in targeting the cellular metabolism for *POLE* mutations, canonical pathways mainly from the KEGG, BIOCARTA, and REACTOME databases as the gene sets were used for GSEA. The results showed that cellular glucose metabolism such as glycolysis and gluconeogenesis were significantly enriched in endometrial cancer samples with *POLE* mutations (Figs. 4a, b). These findings suggest that *POLE* mutations can regulate cellular glucose metabolism in endometrial cancer patients.

In order to further study the function of *POLE* mutations in cellular metabolism through glucose metabolism, we analyzed the expression levels of 20 genes in glucose metabolism and 18 genes in glycolysis in endometrial cancer samples between the *POLE* mutant and the wild type. Endometrial cancer samples were reclassified into two clusters based on the gene expression patterns of these two pathways according to hierarchical clustering. We also examined the mutation rate of *POLE* according to hierarchical clustering. In glucose metabolism, cluster I had a higher gene expression pattern and exhibited a higher rate of *POLE* mutations; yet cluster II had a lower expression pattern and lower rate of *POLE* mutations (Fig. 4c). Therefore, the elevation of glucose metabolism significantly correlated with a higher rate of *POLE* mutations. Similarly, two main groups were also observed in glycolysis. Cluster I had a higher gene expression pattern and exhibited a higher rate of *POLE* mutations, while cluster II had a lower expression pattern and a lower rate of *POLE* mutations (Fig. 4d). Therefore, the elevation of cellular glucose metabolism significantly correlated with a higher rate of *POLE* mutations. These results suggest that the favorable prognosis of *POLE* mutants may be partly due to the regulation of cell metabolism.

### POLE mutations regulate AMF/PGI–AMFR/gp78 expression and patient favorable prognosis

**Fig. 5:**
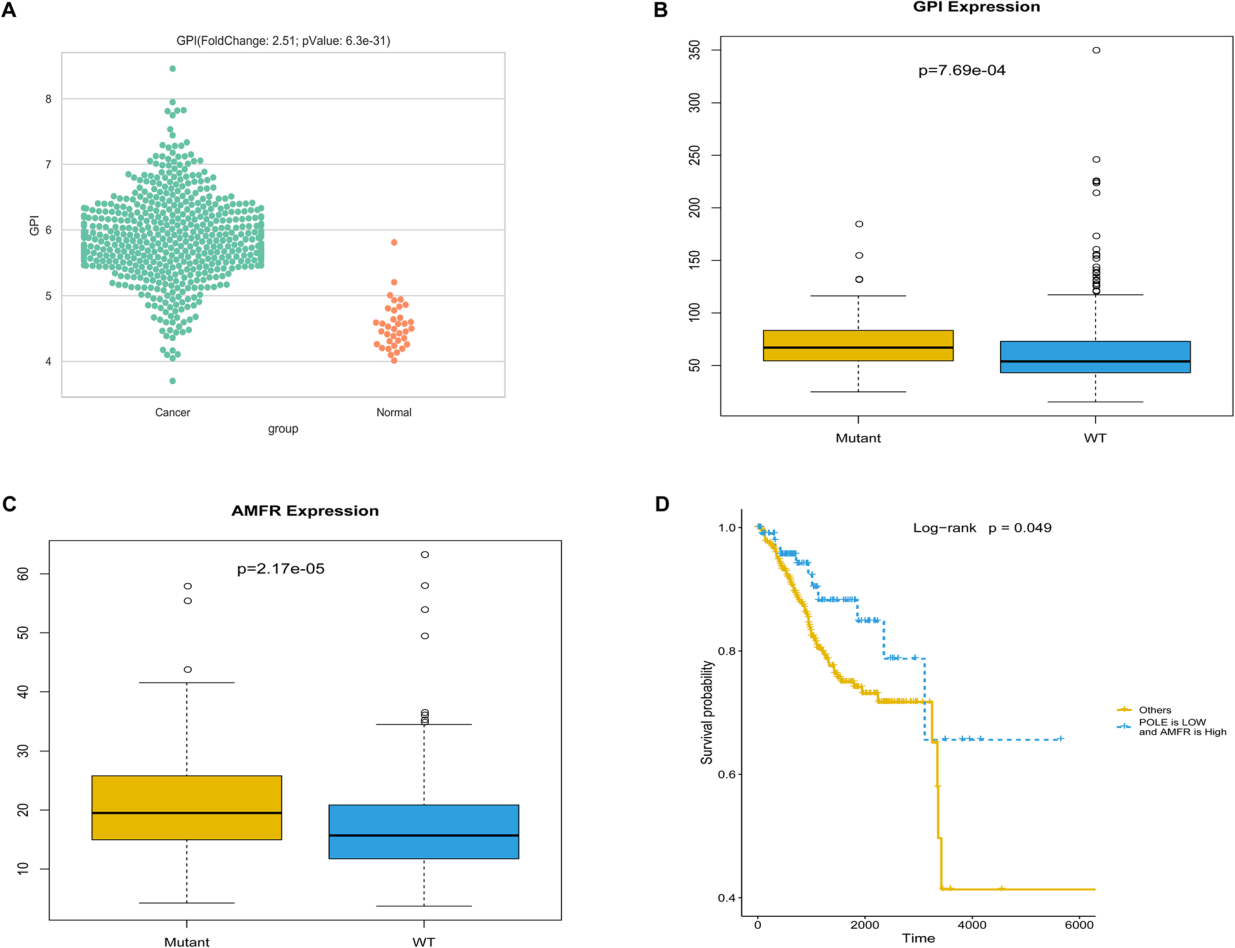
POLE mutations regulate cellular metabolism through enhancing AMF/PGI and AMFR/gp78 expression. **a** The expression of AMF/PGI in endometrial cancer patients and normal samples, Normal = 35, Cancer = 551. The Y axis represents the expression of the gene (log_2_FPKM). **b** The expression of AMF/PGI in mutant and wild type POLE endometrial cancer samples, Mutant = 80, wild type = 446. The Y axis represents the expression of the gene (FPKM). **c** The expression of AMFR/gp78 in mutant and wild type POLE endometrial cancer samples, Mutant = 80, wild type = 446. The Y axis represents the expression of the gene (FPKM). **d** Kaplan–Meier curve and log-rank test for endometrial cancer patients based on the comprehensive expression of POLE and AMFR/gp78 classification

To a certain extent, the results of this study have demonstrated that *POLE* mutations may impact endometrial cancer patient prognosis by regulating cellular glucose metabolism. However, the key molecules involved in the regulation of glucose metabolism by the *POLE* mutations are still unclear. To further target the possible core genes, we analyzed the process of glucose metabolism in detail. Phosphoglucose isomerase (PGI) is a glycolytic enzyme involved in the gluconeogenesis–glycolysis pathways. It is an extracellular cytokine as well, under the name of autocrine motility factor (AMF). Therefore, AMF/PGI plays a dual role as a phosphoglucose isomerase that catalyzes the interconversion of glucose-6-phosphate and fructose-6-phosphatein glycometabolism when it is effective as a cytokine [34–36]. Transcriptomic profiling analysis of 587 endometrial cancer and normal samples revealed differences in the expression of AMF/PGI between endometrial cancer patients and normal samples. In endometrial cancer patients, the expression of AMF/PGI (fold change = 2.51, *P* = 6.3e-31) increased significantly (Fig. 5a). This result suggests that AMF/PGI may play an important role in endometrial cancer. On the other hand, the expression of AMF/PGI has also been detected in POLE mutant and wild type endometrial cancer patients. In endometrial cancer patients with POLE mutations, the expression of AMF/PGI was slightly higher than that in patients with wild type POLE (FC = 1.2, *P* = 0.00077), (Fig. 5b). AMFR/gp78 is a cell surface receptor for AMF/PGI, which is also located in the endoplasmic reticulum where it functions as an E3 ubiquitin ligase. Stimulation of AMFR/gp78 by its ligand AMF/PGI alters cell adhesion, proliferation and apoptosis. AMFR/gp78 expression has been reported in numerous types of human tumors [35, 37]. In POLE mutants and wild type endometrial cancer patients, the expression pattern of AMFR/gp78 was similar as that of AMF/PGI (FC = 1.26, *P* = 0.00002) (Fig. 5c). To further confirm the correlation between POLE mutations and the expression of these two genes, we used the chi-square test to establish the relationship between the POLE mutations and the expression levels of these two genes. The results showed that POLE mutational status was significantly associated with the expression of AMF/PGI (*P* = 7.041e-06) and AMFR/gp78 (*P* = 0.00017).

To further explore the relationship between AMF/PGI or AMFR/gp78 and prognosis of endometrial cancer patients, we analyzed the correlation between the expression levels of these two genes and patient survival by the Kaplan–Meier method and log-rank test. The patient survival curves showed no significant correlation between the expression levels of AMF/PGI or AMFR/gp78, respectively, and the prognosis of endometrial cancer patients (Additional file 1: Figures S4A, B). One interesting result was that the comprehensive expression of POLE and AMFR/gp78 was significantly correlated with patient prognosis. Patient survival curves showed high expressions of AMFR/gp78 and low expression of POLE, which were associated with the favorable prognosis of endometrial cancer patients (Fig. 5d). These results confirm the relationship between POLE mutations, AMF/PGI, AMFR/gp78 expression, and patient prognosis, suggesting that POLE mutations may play a vital role in patient prognosis by affecting the expression and signal transduction of AMF/PGI–AMFR/gp78 as well as affecting cell metabolism.

## Discussion

As a common malignancy in women, the prognosis of endometrial cancer is related to various factors such as tumor type and stage status [38]. In addition, somatic mutations have also been reported to be significantly associated with the prognosis of endometrial cancer such as MUC16 mutations and POLE exonuclease domain mutations [7, 25–27]. Although POLE exonuclease domain mutations have been reported to have a benign effect on prognosis in patients with endometrial cancer, little is known about the relationship and possible mechanism between the POLE mutational status and endometrial cancer patient prognosis. In this study, we identified the existence of somatic POLE mutations in 80 cases of large sporadic individuals in the 530 endometrial cancer samples in the TCGA-UCEC project. We demonstrated that POLE mutations improved prognosis in endometrial cancer patients. The results of the GSEA also confirmed that cellular glucose metabolism was significantly enriched in endometrial cancer patients with POLE mutations, suggesting a possible approach to improving the prognosis of endometrial cancer through POLE mutation.

Accumulating evidence supports that driver mutations and passenger mutations both correlate with cancer progression and clinical outcomes [39, 40]. Consistently, we observed that some somatic mutations were found in many patients including PTEN, PIK3CA, TTN, etc.. Although POLE mutations were present in only 80 of 530 endometrial cancer patients, accounting for 15%, many patients with POLE mutations also carried numerous passenger mutations, resulting in higher mutational burden (Fig. 1c), and these patients showed an improved prognosis (Fig. 2c). Subsequent analysis of the exclusion of these hypermutated cases altered the effects of POLE mutations on patient prognosis and no longer showed a better prognosis. As only 10 POLE mutants were included in this analysis, limited number of samples may lead to insufficient accuracy and reliability of results. Therefore, the effects of higher mutational burden on prognosis and cellular glucose metabolism should be further investigated.

Somatic mutations in POLE have been found in endometrial cancers in many studies. These reports include family studies that have confirmed the existence of novel POLE pathogenic germline variants and phenotypes of POLE-mutations in endometrial cancer (mutation analysis, clinical and lifestyle data, prognosis) [7, 32, 41, 42]. However, how do POLE mutations affect the prognosis of endometrial cancer? One novel objective of this study was to establish the possible relationship between POLE mutations and endometrial cancer prognosis. Our GSEA analyses revealed that cell glucose metabolism might play a vital role in this process. The analysis of DEGs between POLE mutant and wild type also suggests the enrichment of cell metabolism. Collectively, our data demonstrated that endometrial cancer patients with POLE mutations might achieve improved clinical prognosis by regulating cellular glycometabolis. More studies are necessary to investigate the mutant POLE in endometrial cancer therapy and how mutant POLE can be applied to benefit patients.

AMFR/gp78 is a 78 kDa seven-transmembrane glycoprotein which stimulates with AMF/PGI and directs protein kinase C (PKC) activation and receptor phosphorylation. The levels of AMF/PGI and AMFR/gp78 expression are associated with several clinical characteristics including metastasis and prognosis [34, 35, 43–46]. Previously, we have verified that AMF/PGI is abundant at tumor sites to affect the process of tumor growth and metastasis [47], while the expression level of AMF did not relate to the relapse-free survival in endometrial cancer patients [36]. AMF/PGI and AMFR/gp78 are key genes in this process, however there was no significant correlation between the expression of these two genes and the prognosis of endometrial cancer patients, leading to the ambiguous results. The possibility of some other gene expressions that work with AMF/PGI or AMFR/gp78 affecting patient prognosis needs to be clarified. In our results, we found that POLE expression was increased in endometrial cancer patients (FC = 1.65, *P* = 8.1e-15). The expression of POLE also changed slightly between the POLE mutant and the wild type (FC = 1.27, *P* = 2.52e-5). We uncovered that AMF/PGI and AMFR/gp78 both had higher expression in POLE mutants and that the comprehensive low expression of POLE and high expression of AMFR/gp78 showed a positive correlation with patient survival time. POLE may have a synergistic effect on patient outcomes with AMFR/gp78, yet the mechanism needs further study.

## Conclusions

In this study, we utilized whole exome sequencing data, RNA sequencing data, and clinical information in the TCGA-UCEC project downloaded from the TCGA database for comprehensive analysis. The results suggest that POLE mutations can significantly improve the prognosis of endometrial cancer patients and 15% of patients (Number = 80) possessed POLE somatic mutations. The differential expressed genes between the POLE mutant and the wild type patients indicated the enrichment of glycometabolis Clustering analysis showed a higher expression of glucose metabolism genes and a higher rate of POLE mutations. Survival analysis through the Kaplan–Meier method unraveled the relationship between the comprehensive expression of POLE, AMFR/gp78 and patient prognosis.

Taken together, POLE mutations improve the prognosis of endometrial cancer via regulating cellular metabolism through AMF/AMFR signal transduction. These results may provide novel and promising approaches for the clinical treatment of endometrial cancer. More in-depth research is needed to validate our findings and explore the feasibility of the developed method in clinical application.

## Supplementary information

**Additional file 1: Table S1.** The number of mutation classification and type in 530 endometrial cancer patients. **Table S2.** The mutation frequency of top 124 mutated genes in 530 endometrial cancer patients. **Table S3.** The relationship between *POLE* mutations and four clinical characteristics by chi-square test. **Figure S1.** SNP and mutation istatistics in 530 endometrial cancer patients. **Figure S2.** Kaplan-Meier curve and Log-rank test for endometrial cancer patients based on POLE mutational status classification when excluded hypermutated phenotypes (more than 500 mutations per sample), *N* = 387. **Figure S3.** (A-B) GSEA of genes between mutant and wild type POLE endometrial cancer samples. **Figure S4.** (A) Kaplan-Meier curve and Log-rank test for endometrial cancer patients based on expression level of AMF/GPI classification. (B) Kaplan-Meier curve and Log-rank test for endometrial cancer patients based on expression level of AMFR/gp78 classification.

## Data Availability

All data used or analysed during this study are provided in TCGA database (https://cancergenome.nih.gov/), and the TCGA-UCEC project (https://portal.gdc.cancer.gov/projects/TCGA-UCEC).

## Abbreviations

AMF: Autocrine motility factor
DEL: Short deletions
EC: Endometrial cancer
INS: Short insertions
PGI: Phosphoglucose isomerase
POLE: DNA polymerase ε
SNPs: Single-nucleotide polymorphisms
TCGA: The Cancer Genome Atlas
UCEC: Uterine Corpus Endometrial Carcinoma
