# Supplementary material for: POLE mutations improve the prognosis of endometrial cancer via regulating cellular metabolism through AMF/AMFR signal transduction
Source: BMC Med Genet. 2019 Dec 21;20:202. doi: 10.1186/s12881-019-0936-2 (PMC6925869; doi:10.1186/s12881-019-0936-2)
Supplement: Supplementary file 1 — Additional file 1: Table S1. The number of mutation classification and type in 530 endometrial cancer patients. Table S2. The mutation frequency of top 124 mutated genes in 530 endometrial cancer patients. Table S3. The relationship between POLE mutations and four clinical characteristics by chi-square test. Figure S1. SNP and mutation istatistics in 530 endometrial cancer patients. Figure S2. Kaplan-Meier curve and Log-rank test for endometrial cancer patients based on POLE mutational status classification when excluded hypermutated phenotypes (more than 500 mutations per sample), N = 387. Figure S3. (A-B) GSEA of genes between mutant and wild type POLE endometrial cancer samples. Figure S4. (A) Kaplan-Meier curve and Log-rank test for endometrial cancer patients based on expression level of AMF/GPI classification. (B) Kaplan-Meier curve and Log-rank test for endometrial cancer patients based on expression level of AMFR/gp78 classification. [file 12881_2019_936_MOESM1_ESM.docx]

| **Type** | **All** | **Synonymous variants (including silent mutations)** | **Non-synonymous variants** |
| --- | --- | --- | --- |
| DEL | 41112 | 21695 | 19417 |
| INS | 23292 | 8452 | 14840 |
| SNPs | 821967 | 357513 | 464454 |

Table S1. The number of mutation classification and type in 530 endometrial cancer patients.

| **Synonymous variants (including silent mutations): 387660** | | **Non-synonymous variants: 498711** | |
| --- | --- | --- | --- |
| Type | Number | Type | Number |
| Silent | 150337 | Frame_Shift_Del | 18527 |
| RNA | 18548 | Frame_Shift_Ins | 10951 |
| Intron | 48333 | In_Frame_Del | 638 |
| 5'Flank | 6642 | In_Frame_Ins | 1967 |
| 5'UTR | 18579 | Missense_Mutation | 415573 |
| 3'UTR | 125559 | Nonsense_Mutation | 40375 |
| 3'Flank | 12287 | Nonstop_Mutation | 394 |
| Splice_Region | 7252 | Splice_Site | 9906 |
| IGR | 123 | Translation_Start_Site | 380 |

Table S2. The mutation frequency of top 124 mutated genes in 530 endometrial cancer patients.

| Gene Symbol | No. of Mutated Samples | Total No. of Samples | Mutation sample proportion |
| --- | --- | --- | --- |
| PTEN | 304 | 530 | 57.36% |
| PIK3CA | 253 | 530 | 47.74% |
| TTN | 233 | 530 | 43.96% |
| ARID1A | 226 | 530 | 42.64% |
| TP53 | 191 | 530 | 36.04% |
| MUC16 | 159 | 530 | 30.00% |
| PIK3R1 | 158 | 530 | 29.81% |
| KMT2D | 141 | 530 | 26.60% |
| CTCF | 129 | 530 | 24.34% |
| CSMD3 | 128 | 530 | 24.15% |
| ZFHX3 | 126 | 530 | 23.77% |
| CTNNB1 | 125 | 530 | 23.58% |
| RYR2 | 123 | 530 | 23.21% |
| SYNE1 | 121 | 530 | 22.83% |
| MUC5B | 120 | 530 | 22.64% |
| KMT2B | 119 | 530 | 22.45% |
| FAT4 | 116 | 530 | 21.89% |
| ZFHX4 | 116 | 530 | 21.89% |
| OBSCN | 115 | 530 | 21.70% |
| PCLO | 115 | 530 | 21.70% |
| NEB | 114 | 530 | 21.51% |
| MACF1 | 111 | 530 | 20.94% |
| FLG | 111 | 530 | 20.94% |
| USH2A | 110 | 530 | 20.75% |
| LRP1B | 109 | 530 | 20.57% |
| DNAH7 | 109 | 530 | 20.57% |
| CCDC168 | 107 | 530 | 20.19% |
| DNAH5 | 107 | 530 | 20.19% |
| FAT3 | 107 | 530 | 20.19% |
| CHD4 | 107 | 530 | 20.19% |
| FAT1 | 106 | 530 | 20.00% |
| DMD | 105 | 530 | 19.81% |
| RYR3 | 103 | 530 | 19.43% |
| DST | 102 | 530 | 19.25% |
| LRP2 | 101 | 530 | 19.06% |
| TAF1 | 101 | 530 | 19.06% |
| ADGRV1 | 100 | 530 | 18.87% |
| HMCN1 | 99 | 530 | 18.68% |
| HERC2 | 99 | 530 | 18.68% |
| RYR1 | 99 | 530 | 18.68% |
| ARHGAP35 | 99 | 530 | 18.68% |
| DNAH8 | 98 | 530 | 18.49% |
| DNAH10 | 98 | 530 | 18.49% |
| HUWE1 | 98 | 530 | 18.49% |
| APOB | 98 | 530 | 18.49% |
| CSMD1 | 97 | 530 | 18.30% |
| AHNAK2 | 97 | 530 | 18.30% |
| FAT2 | 97 | 530 | 18.30% |
| TENM1 | 97 | 530 | 18.30% |
| KRAS | 97 | 530 | 18.30% |
| ABCA13 | 96 | 530 | 18.11% |
| MDN1 | 96 | 530 | 18.11% |
| FBXW7 | 96 | 530 | 18.11% |
| PCDH15 | 95 | 530 | 17.92% |
| ZNF292 | 95 | 530 | 17.92% |
| DNAH11 | 94 | 530 | 17.74% |
| KMT2C | 94 | 530 | 17.74% |
| NSD1 | 94 | 530 | 17.74% |
| ANK3 | 93 | 530 | 17.55% |
| MED12 | 93 | 530 | 17.55% |
| AHNAK | 92 | 530 | 17.36% |
| SSPO | 92 | 530 | 17.36% |
| ATM | 92 | 530 | 17.36% |
| SYNE2 | 91 | 530 | 17.17% |
| CACNA1E | 91 | 530 | 17.17% |
| MUC4 | 91 | 530 | 17.17% |
| MKI67 | 90 | 530 | 16.98% |
| PRKDC | 90 | 530 | 16.98% |
| LAMA2 | 90 | 530 | 16.98% |
| DYNC2H1 | 89 | 530 | 16.79% |
| NBEA | 89 | 530 | 16.79% |
| SPTA1 | 89 | 530 | 16.79% |
| ADGRG4 | 88 | 530 | 16.60% |
| LRP1 | 88 | 530 | 16.60% |
| UBR4 | 88 | 530 | 16.60% |
| JAK1 | 88 | 530 | 16.60% |
| SETD1B | 88 | 530 | 16.60% |
| SACS | 87 | 530 | 16.42% |
| DNAH9 | 87 | 530 | 16.42% |
| DNAH2 | 87 | 530 | 16.42% |
| PKHD1L1 | 87 | 530 | 16.42% |
| TG | 87 | 530 | 16.42% |
| CEP290 | 87 | 530 | 16.42% |
| XIRP2 | 86 | 530 | 16.23% |
| MUC17 | 86 | 530 | 16.23% |
| SZT2 | 86 | 530 | 16.23% |
| ABCA12 | 85 | 530 | 16.04% |
| HERC1 | 85 | 530 | 16.04% |
| WDFY3 | 85 | 530 | 16.04% |
| ASPM | 84 | 530 | 15.85% |
| EYS | 84 | 530 | 15.85% |
| AKAP9 | 84 | 530 | 15.85% |
| DYNC1H1 | 84 | 530 | 15.85% |
| USP9X | 84 | 530 | 15.85% |
| CSMD2 | 84 | 530 | 15.85% |
| MGA | 84 | 530 | 15.85% |
| WDR87 | 83 | 530 | 15.66% |
| COL6A3 | 83 | 530 | 15.66% |
| DCHS1 | 83 | 530 | 15.66% |
| BCOR | 83 | 530 | 15.66% |
| DNAH3 | 82 | 530 | 15.47% |
| RNF213 | 82 | 530 | 15.47% |
| SVEP1 | 82 | 530 | 15.47% |
| USP34 | 82 | 530 | 15.47% |
| SI | 82 | 530 | 15.47% |
| COL11A1 | 82 | 530 | 15.47% |
| NIPBL | 82 | 530 | 15.47% |
| PPP2R1A | 82 | 530 | 15.47% |
| KIAA1109 | 81 | 530 | 15.28% |
| BIRC6 | 81 | 530 | 15.28% |
| PKHD1 | 81 | 530 | 15.28% |
| ANK2 | 81 | 530 | 15.28% |
| SMG1 | 81 | 530 | 15.28% |
| WDFY4 | 81 | 530 | 15.28% |
| FRAS1 | 81 | 530 | 15.28% |
| CHD3 | 81 | 530 | 15.28% |
| RNF43 | 81 | 530 | 15.28% |
| DCHS2 | 80 | 530 | 15.09% |
| LRRK2 | 80 | 530 | 15.09% |
| FSIP2 | 80 | 530 | 15.09% |
| UTRN | 80 | 530 | 15.09% |
| FCGBP | 80 | 530 | 15.09% |
| POLE | 80 | 530 | 15.09% |

Table S3. The relationship between *POLE* mutations and four clinical characteristics by chi-square test.

| chi-square test | Histological type | Histologic grade | Clinical stage | Age at initial pathologic diagnosis |
| --- | --- | --- | --- | --- |
| POLE type | 0.0006766 | 0.7495 | 1 | 4.39E-05 |


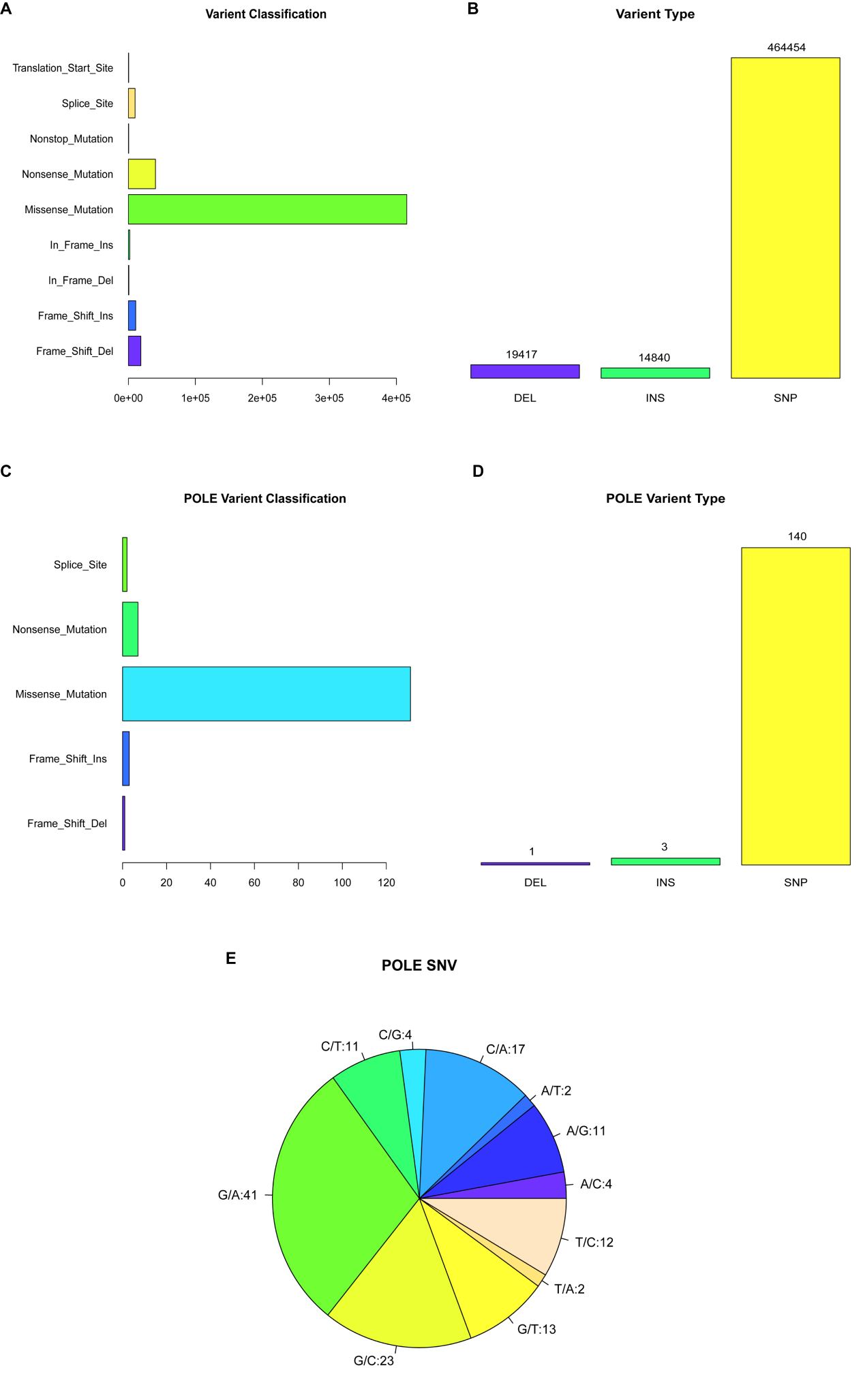


Figure S1. SNP and mutation istatistics in 530 endometrial cancer patients.

1. The number of somatic mutations based on variant classification.
2. The number of somatic mutations based on variant type.
3. The number of somatic mutations of *POLE* based on variant classification.
4. The number of somatic mutations of *POLE* based on variant type.

(E)The pie chart of mutations of POLE based on single nucleotide variant (SNV) class.


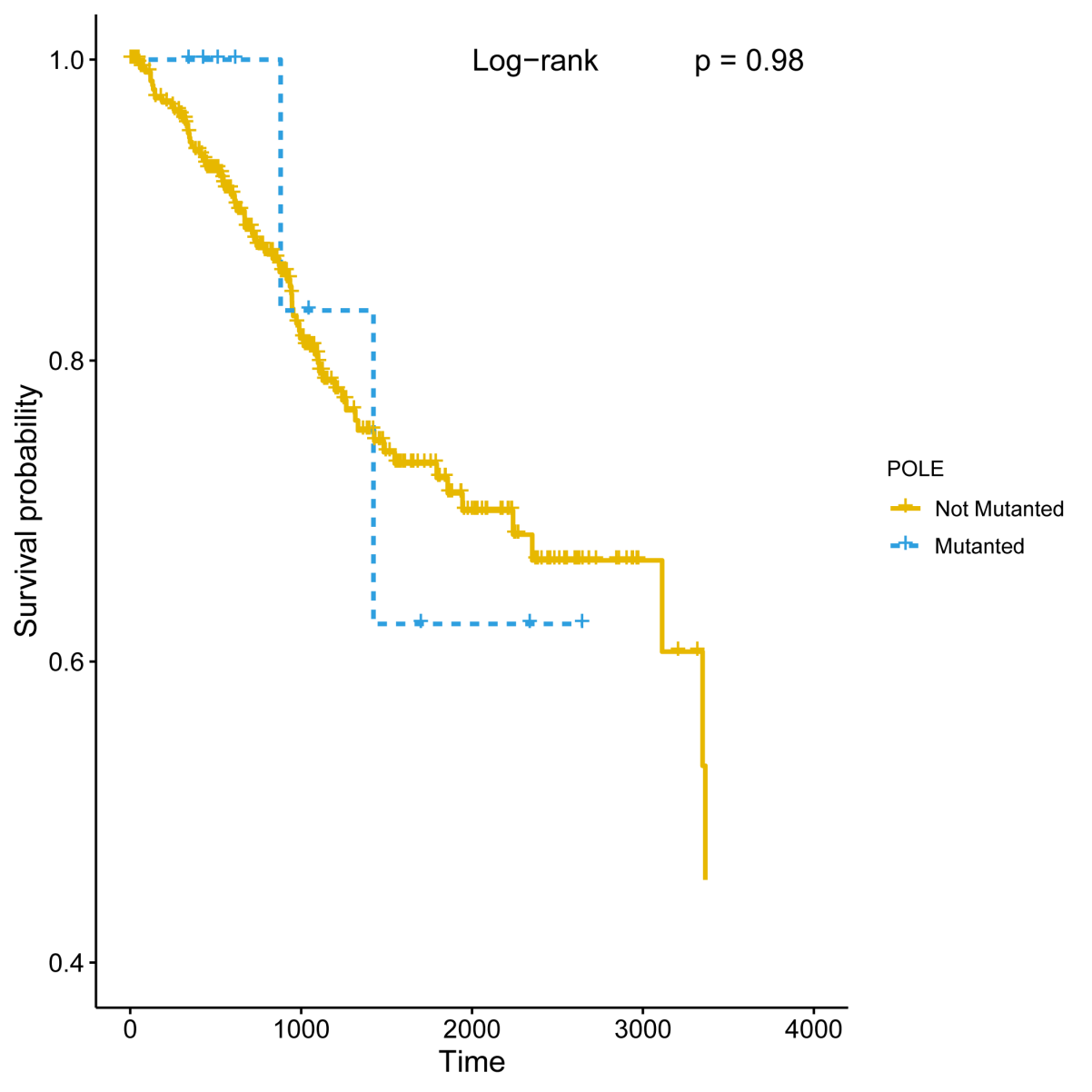


Figure S2. Kaplan-Meier curve and Log-rank test for endometrial cancer patients based on POLE mutational status classification when excluded hypermutated phenotypes (more than 500 mutations per sample), N = 387.


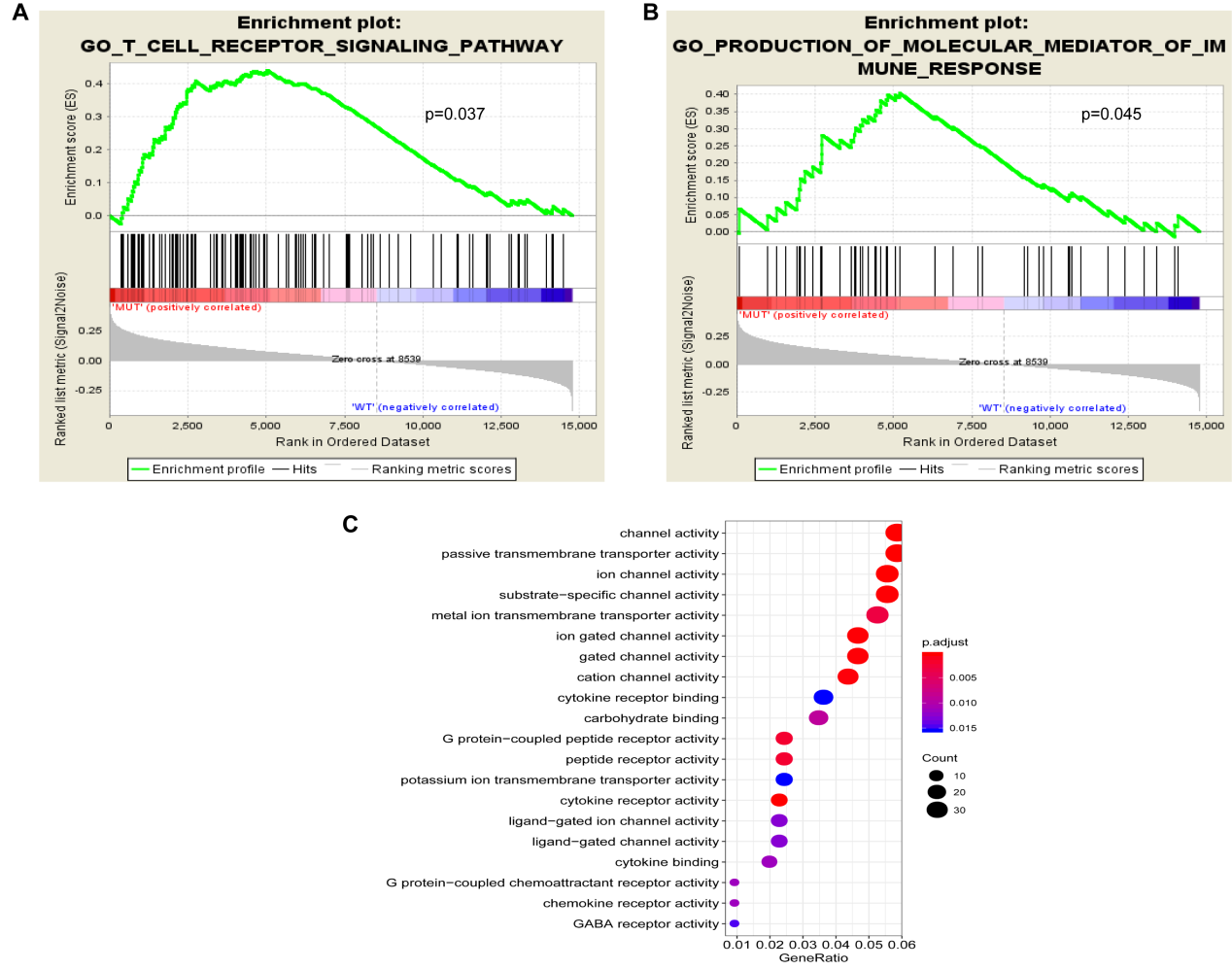


Figure S3. (A-B) GSEA of genes between mutant and wild type POLE endometrial cancer samples. Gene sets annotated by GO terms were used in the analysis. Gene sets representing T cell receptor signaling pathway (A) and production of molecular mediator of immune response (B) were significantly enriched. (C) Molecular function analysis of DEGs *via* hypergeometric algorithm. The dot color represents the *P* value and the dot size represents the number of DEGs.


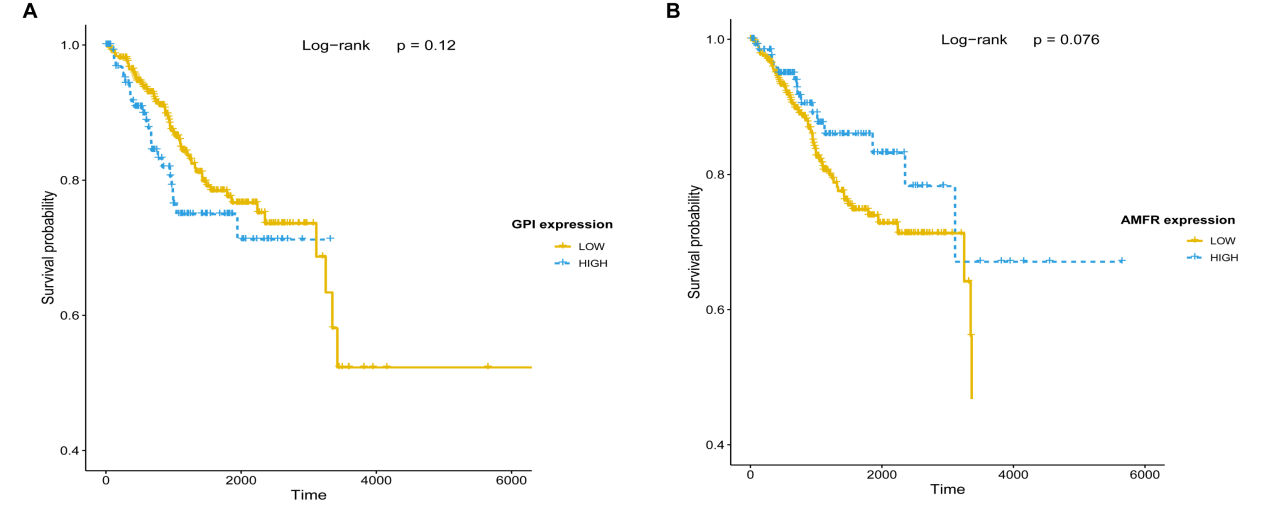


Figure S4. (A) Kaplan-Meier curve and Log-rank test for endometrial cancer patients based on expression level of AMF/GPI classification. (B) Kaplan-Meier curve and Log-rank test for endometrial cancer patients based on expression level of AMFR/gp78 classification.
